# Establishment of a Recombinant AAV2/HBoV1 Vector Production System in Insect Cells

**DOI:** 10.3390/genes11040439

**Published:** 2020-04-17

**Authors:** Xuefeng Deng, Wei Zou, Ziying Yan, Jianming Qiu

**Affiliations:** 1 Department of Microbiology, Molecular Genetics and Immunology, University of Kansas Medical Center, Kansas City, KS 66160, USA; xuefeng2251@gmail.com (X.D.); zouw@umich.edu (W.Z.); 2 Department of Anatomy and Cell Biology, University of Iowa, Iowa City, IA 52242, USA; ziying-yan@uiowa.edu

**Keywords:** rAAV2/HBoV1, baculovirus, insect cells

## Abstract

We have previously developed an rAAV2/HBoV1 vector in which a recombinant adeno-associated virus 2 (rAAV2) genome is pseudopackaged into a human bocavirus 1 (HBoV1) capsid. Recently, the production of rAAV2/HBoV1 in human embryonic kidney (HEK) 293 cells has been greatly improved in the absence of any HBoV1 nonstructural proteins (NS). This NS-free production system yields over 16-fold more vectors than the original production system that necessitates NS expression. The production of rAAV with infection of baculovirus expression vector (BEV) in the suspension culture of Sf9 insect cells is highly efficient and scalable. Since the replication of the rAAV2 genome in the BEV system is well established, we aimed to develop a BEV system to produce the rAAV2/HBoV1 vector in Sf9 cells. We optimized the usage of translation initiation signals of the HBoV1 capsid proteins (Cap), and constructed a BEV Bac-AAV2Rep-HBoV1Cap, which expresses the AAV2 Rep78 and Rep52 as well as the HBoV1 VP1, VP2, and VP3 at the appropriate ratios. We found that it is sufficient as a trans helper to the production of rAAV2/HBoV1 in Sf9 cells that were co-infected with the transfer Bac-AAV2ITR-GFP-luc that carried a 5.4-kb oversized rAAV2 genome with dual reporters. Further study found that incorporation of an HBoV1 small NS, NP1, in the system maximized the viral DNA replication and thus the rAAV2/HBoV1 vector production at a level similar to that of the rAAV2 vector in Sf9 cells. However, the transduction potency of the rAAV2/HBoV1 vector produced from BEV-infected Sf9 cells was 5–7-fold lower in polarized human airway epithelia than that packaged in HEK293 cells. Transmission electron microscopy analysis found that the vector produced in Sf9 cells had a high percentage of empty capsids, suggesting the pseudopackage of the rAAV2 genome in HBoV1 capsid is not as efficient as in the capsids of AAV2. Nevertheless, our study demonstrated that the rAAV2/HBoV1 can be produced in insect cells with BEVs at a comparable yield to rAAV, and that the highly efficient expression of the HBoV1 capsid proteins warrants further optimization.

## 1. Introduction

Adeno-associated virus (AAV) and human bocavirus (HBoV) are members in different genera of the parvovirus family [1]. AAV is a nonpathogenic parvovirus and its productive replication needs the function of a helper virus [2,3]. In contrast, HBoV1 is a human pathogen that causes lower respiratory tract infections in young children worldwide [4,5,6,7,8,9,10,11,12]. In vitro, HBoV1 infects only polarized human airway epithelium cultured at an air–liquid interface (HAE-ALI), and replicates autonomously [13,14,15,16]. While both are nonenveloped, small, single-stranded (ss) DNA viruses, AAV packages both the plus- and minus-strand genome with equal efficiency, whereas HBoV1 prefers packaging the minus-strand [14]. HBoV1 has a genome of 5543 nucleotides (nts) in length, which is 18.5% (864 nts) larger than the AAV2 genome of 4679 nts [14].

The genome organizations of these two viruses are quite different. HBoV1 uses one promoter to express all viral nonstructural (NS) and structural or capsid (Cap) proteins, but AAV uses three different promoters [4,17]. The coding sequence of AAV consists of two large open reading frames (ORFs) encoding the nonstructural or replication (Rep) proteins and the Cap proteins at the left and right half of the AAV genome, respectively [18]. The large Rep78/68 and the small Rep52/40 proteins are expressed from the viral mRNAs that are transcribed by an upstream promoter at unit 5 of the genome (P5) and an internal promoter (P19), respectively. Three AAV Cap proteins, VP1, VP2 and VP3, are expressed from the mRNA transcribed by the P40 promoter [2,19]. In addition, a small NS protein, assembly-associated protein (AAP), is alternatively translated from the P40-transcribed Cap-coding mRNA [20], which plays a role in capsid assembly [21,22,23,24]. Recently, another small NS protein, membrane-associated accessory protein (MAAP), has been identified, which is also expressed from the P40-transcribed mRNA through alternative translation [25]. It exists in all AAV serotypes and was believed to play a role in the life cycle of AAV. HBoV1 expresses five NS proteins, NS1, NS2, NS3, NS4, and nuclear protein (NP1), and three Cap proteins, VP1, VP2, and VP3, as well as a bocaviral noncoding small RNA (BocaSR) [26,27,28,29]. The middle ORF, which is located at the center of the genome between the left ORF encoding NS1-4 and the right ORF encoding VP1-3, encodes NP1 [4]. NP1 plays an important role in viral DNA replication [30], as well as in regulation of HBoV1 cap expression [28,31].

The sequences at both termini of AAV and HBoV1 contain important motifs that are necessary for viral genome replication and virion assembly. In AAV, they are inverted terminal repeats (ITRs) [2,19], but in HBoV1 they are asymmetric with a non-perfectly palindromic hairpin at the left end terminus and a perfectly palindromic hairpin at the right terminus [14]. The transfection of the plasmid clone of a complete AAV genome in human embryonic kidney (HEK)293 cells leads to the production of AAV virions, but this only occurs in the presence of infection of a helper virus, such as adenovirus or co-transfection of a plasmid helper harboring all the adenoviral helper genes (*E2*, *E4Orf6*, and *VA RNA*) [32]. While HEK293 cells are not permissive to HBoV1 infection, the transfection of the cloned HBoV1 genome can produce HBoV1 infectious virions. The progeny virions infect HAE-ALI with a robustly high efficiency, even at a multiplicity of infection (MOI) of at 0.001 viral genome per cell [14,15].

Trans-complementation supports the replication and package of the gutless rAAV2 genome containing only cis elements of their termini and a gene of interest, which have been effectively developed as rAAV vectors for gene therapy of genetic diseases [2,19,33]. The safety profiles of the rAAV genome have been proven from tremendous preclinical studies and clinical trials of human gene therapy [34,35,36,37,38,39,40,41]. Up to date, two rAAV-based drugs, Luxturna and Zolgensma, have been approved by the US FDA. Similarly, a recombinant HBoV1 vector (rHBoV1) was produced in HEK293 cells via trans-complementation [42]. rHBoV1 efficiently transduced HAE-ALI from the apical membrane; however, the safety concern of being derived from a human pathogen limits its application. To overcome this disadvantage for a safe vector to transduce human airway epithelium from the airway lumen with the emphasis on gene therapy for cystic fibrosis (CF) lung disease, we successfully developed a cross-genera chimeric parvovirus vector, rAAV2/HBoV1 [42], in which the safety-proven rAAV2 genome was packaged into the airway tropic HBoV1 capsid. Importantly, the rAAV2/HBoV1 expands the package capacity of the rAAV2 genome by 20%, up to 5.8-kb [42]. Apical application of an rAAV2/HBoV1 carrying a full-length CF transmembrane conductance regulator (CFTR) cDNA of 5.4-kb to CF HAE-ALI cultures, which were made of primary airway epithelial cells of CF patients, efficiently corrected CFTR-dependent chloride transport [42]. In addition, the rAAV2/HBoV1 vector efficiently transduced ferret airways in vivo [43]. Therefore, it holds much promise for gene delivery to human airways, as well as for preclinical trials of CF gene therapy using CF ferret models [44]. Recently we have increased the production efficiency of the rAAV2/HBoV1 vector in HEK293 cells through optimization of cap expression, which approaches a similar level of rAAV2 production in HEK293 cells [45]. However, a robust vector production system is in demand for future CF gene therapy in preclinical and human trials using the rAAV2/HBoV1 vector.

Traditional rAAV vector productions utilize HEK293 cells. During the rAAV2 or the rAAV2/HBoV1 production in HEK293 cells, the rescue and replication of the rAAV2 genome require the expression of AAV *rep* in addition to the adenoviral helper genes [19,46]. rAAV2 can also be produced in insect cells by the infection of baculovirus expression vectors (BEVs). The AAV-BEV production system represents a robust and scalable bioprocess [47,48,49,50,51,52], which only requires one of the large Rep78/68 and one of the small Rep52/40 [53]. AAP is required for efficient production of certain serotypes of rAAV vectors in Sf9 cells [54,55]. Co-infection of BEVs—one carrying an rAAV2 genome and one expressing AAV2 Rep78 and Rep52 along with AAV2 VP1, VP2, and VP3—has been used to produce the rAAV vector in a large quantity at a yield of up to ~10^5^ copies per Sf9 cell, compared to the yield of ~10^3^ copies per HEK293 cell [47,53,54,56].

In this report, we explored the possibility of rAAV2/HBoV1 vector production in the BEV system. Our study demonstrated that the rAAV2/HBoV1 vector can be efficiently produced in a suspension Sf9 culture. In the presence of the expression of HBoV1 NP1, a vector yield similar to that of rAAV2 was achieved in Sf9 cells. To our knowledge, this is the first report that the parvoviral cross-genera pseudopackage is also effective in insect cells.

## 2. Materials and Methods

### 2.1. Cell and Cell Culture

Human embryonic kidney (HEK) 293 cells: HEK293 cells (CRL-1573), obtained from American Type Culture Collection (ATCC; Manassas, VA, USA), were cultured in Dulbecco’s modified Eagle’s medium (GE Healthcare Life Sciences, Piscataway, NJ, USA) with 10% fetal bovine serum (#F0926, MilliporeSigma, St. Louis, MO, USA)

Insect cells: Sf9 cells (CRL-1711, ATCC) were cultured in suspension in SFX-Insect medium (GE Healthcare, Marlborough, MA, USA) with 2% fetal bovine serum (#F0926, Millipore Sigma; St. Louis, MO, USA) at 27 °C.

HAE-ALI cultures: primary human airway cells were isolated from human lung tissues, and this procedure was carried out at the Tissue and Cell Culture Core of the Center for Gene Therapy, University of Iowa. The primary cells were cultured in the airway basal cell expansion medium (#CC-3118, Lonza, Basel, Switzerland), supplemented with 10 µM of ROCK inhibitor Y-276322, 1 µM of A8301, 1 µM of DMH-1, and 1 µM of CHIR99021 (Tocris Biosciences, Minneapolis, MN, USA) until confluency [57]. Then, the cells were collected and seeded onto collagen-coated inserts (Transwells; #3470, Corning, Tewksbury, MA, USA) with a density of 50,000 cells/well. After cell attachment for two days, the apical and basolateral medium were removed and replaced with a complete Pneumacult-ALI medium (StemCell, Vancouver, Canada) at the basolateral chamber to initiate an airway–liquid interface. The medium was changed every two days, and the ALI-cultured HAE took about four weeks to be fully differentiated. We monitored the cultures with a transepithelial electrical resistance using an epithelial Ohmvoltmeter (Millicell-ERS; EMD-Millipore, Burlington, MA, USA), and only HAE-ALI cultures with a resistance value of over 1000 Ω·cm^2^ were used for subsequent transduction.

### 2.2. Construction of Baculoviral Expression Shuttle Plasmids and Other HEK293 Cell-Expressing Plasmids

pFastBacDual(m): the plasmid pFastBacDual (Invitrogen, Carlsbad, CA, USA) was modified by inserting a 0.83-kb fragment of λ DNA which contains a SbfI site at each end through SnaBI at nt 3983 and MfeI at nt 4815 to obtain the plasmid pFastBacDual (m).

**Figure 1:**
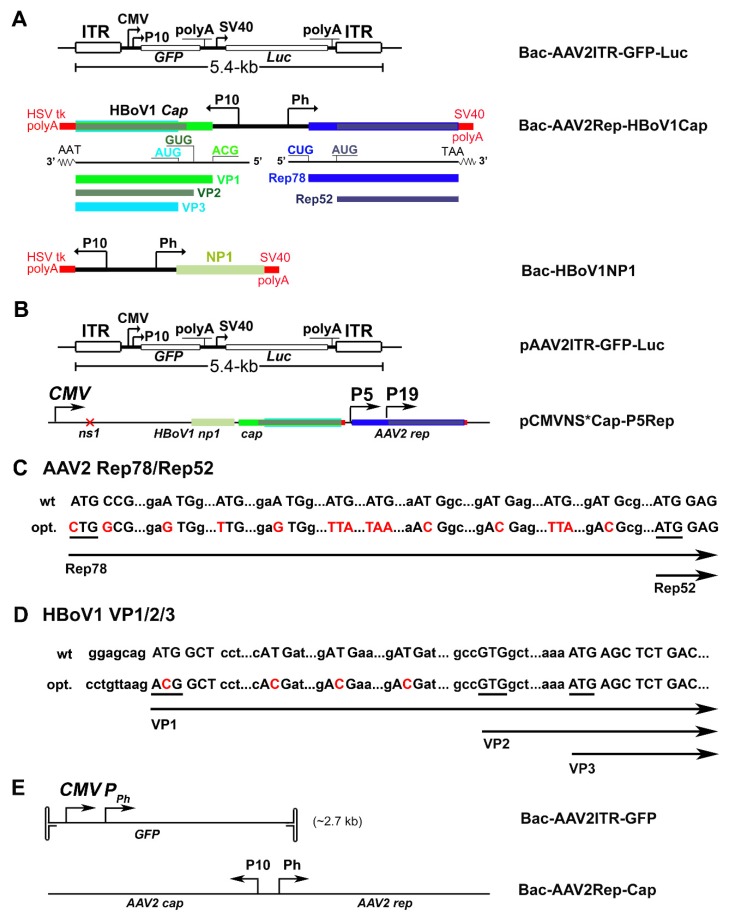
Construction of baculoviral transfer plasmids for vector production in Sf9 cells and the plasmids for rAAV2/HBoV1 vector production in HEK293 cells. (**A**) BEVs for rAAV2/HBoV1 production. Schematically diagrammed are structures inside the BEVs that were involved in rAAV2/HBoV1 production. Bac-AAV2ITR-GFP-Luc carries an rAAV2 genome of 5.4-kb; Bac-AAV2Rep-HBoV1Cap expresses AAV2 Rep proteins and HBoV1 capsid proteins as shown; and Bac-HBoV1NP1 expresses HBoV1 NP1. P10 and Ph are baculoviral promoters, and CMV and SV40 are cytomegaloviral immediate early and SV40 virus early promoters, respectively. PolyA: polyadenylation signal; Luc: firefly luciferase. (**B**) Plasmids used for vector production in HEK293 cells. pAAV2ITR-GFP-Luc carries the same rAAV2 genome as shown in panel A. pCMVNS*Cap-P5Cap is a two-in-one plasmid. It was derived from the plasmid pHBoV1CMVNS*Cap [28], in which the NS1/2 ORF was early terminated. An AAV2 P5 and P19 driven *rep* gene was cloned after the CMV promoter-driven HBoV1 *cap* gene expression cassette. (**C**,**D**) Codon optimization. Both wild type (wt) and optimized (opt.) sequences between ATGs of the AAV2 Rep78 and Rep52 ORFs (**C**) and of the HBoV1 VP1 and VP3 ORFs (**D**) are diagrammed. Nucleotides in red indicate mutations. (**E**) BEVs for rAAV2 production in Sf9 cells. Bac-AAV2ITR-GFP carries a GFP expression cassette under both the CMV and P10 promoters. Bac-AAV2Rep-Cap carries expression cassettes of AAV2 *cap* and AAV2 *rep* under the P10 and Ph promoters, respectively.

pBac-AAV2ITR-GFP-Luc (5.4-kb): this BAC-AAV transfer plasmid was constructed by replacing the 0.83-kb λ DNA in pFastBacDual(m) with a 5444-nt ITR-flanked (rAAV) proviral DNA into the at two SbfI sites (Figure 1A). The intermediate rAAV proviral plasmid pAAV-CMV(P10)-GFP-SV40-Luc-bGHpA was derived from pAAV-MCS vector (Cell Biolabs, Inc., San Diego, CA, USA). Foreign DNA flanking with a pair of SbfI sites was cloned into EcoRI and BamHI digested pAAV-MCS, including the P10 promoter, an open reading frame (ORF) of an enhanced green fluorescent protein (GFP; excised from pEGFP1, Clontech, Palo Alto, CA, USA), SV40 polyadenylation signal (polyA), SV40 early promoter, a FLAG-tagged ORF of firefly luciferase (Luc) and a stuffer from λ DNA (to make the rAAV2 genome of 5444-nt).

pBac-AAV2Rep-HBoV1Cap: to obtain a modified AAV2 *rep* gene expression cassette of a bifunctional Rep78- and Rep52-encoding mRNA, we synthesized a 637-bp DNA fragment containing a partially codon-optimized (opt)Rep78 ORF [56] (Figure 1C), and amplified the full-length optRep78/52 ORF using overlapped PCR, which was cloned into pFastBacDual through BglII (BamHI)-XbaI sites and resulted in pFastBacDual-AAV2Rep. We also synthesized a fragment of 390-bp containing an optimized HBoV1 sequence between VP1 AUG and VP3 AUG [28,58], as shown in Figure 1D, and amplified the full length optVP1/2/3 ORF using overlapped PCR, which was then cloned into the pFastBacDual-AAV2Rep through XhoI-NheI sites to obtain the BEV transfer plasmid pBac-AAV2Rep-HBoV1Cap (Figure 1A).

pBac-HBoV1NP1: HBoV1 NP1 ORF was cloned into pFastBacDual between the XhoI-KpnI sites to get the transfer construct pBac-HBoV1-NP1 (Figure 1A).

pAAV2ITR-GFP-Luc: to parallel compare the capability of the Sf9 cell and HEK293 cell systems, we made a plasmid, pAAV2ITR-GFP-Luc (Figure 1B), based on the backbone of pAAV-MCS promoterless (Cell Biolabs). This was achieved by cloning the fragments from the plasmid of pFastBacDual(m)-AAV2-ITR-GFP-Luc through two NotI sites.

pCMVNS*Cap-P5Rep: this was the HBoV1 *cap* and AAV2 *rep* expression two-in-one helper plasmid (Figure 1B), which was constructed by cloning the P5- and P19-driven AAV2 *rep* expression cassette from the pAV-Rep2 plasmid [42] into the pCMVNS*Cap [28], which expresses HBoV1 capsid proteins under the cytomegalovirus immediate early promoter (CMV) through a SacII site.

All the plasmids were sequenced to confirm the expressing genes and the critical elements for virus production at MCLAB (South San Francisco, CA, USA).

### 2.3. Recombinant Baculovirus Expression vector (BEV) Production

BEVs were generated by transfection of the BEV shuttle plasmid into DH10Bac^TM^ *E. coli* competent cells, following the instructions of the Bac-to-Bac Baculovirus Expression System (Invitrogen, Carlsbad, CA). Bac-AAV2ITR-GFP (Bac-GFP) [47] and Bac-AAV2Rep-Cap (Bac-RepCap2) [56] (Figure 1E) were obtained from The University of Iowa Viral Vector Core Facility. BEVs were titrated in plaque forming units (pfu) by a plaque assay as described in the manual of the Bac-to-Bac Baculovirus Expression System (Invitrogen) or quantified using quantitative real-time PCR (qPCR) with an amplicon targeting to the gentamicin-resistance gene (Probe: 5′ 6FAM-ACA TTC ATC GCG CTT GCT GCC TTC-3′ ZEN /Iowa Black FQ; forward primer: 5′-CGG GAA CTT GCT CCG TAG TAA-3′, and reverse primer: 5′-CGC CAA CAA CCG CTT CTT-3′).

### 2.4. rAAV vector Production

For production of AAV vectors in insect cells, 200 mL of Sf9 cells in suspension culture at a density of 2 × 10^6^ cells/mL were co-infected with BEVs at an MOI of ~10 (pfu/cell). At 72 hrs post-infection, cells were collected by centrifugation and lysed in phosphate-buffered saline, pH7.4 (PBS). After four times of freezing-thawing, the cells were sonicated at the setting of 70% power for 3 min (1 min sonicate with 1 min of interval), followed by DNase I treatment at 37 °C for 45 min and 10% deoxycholate with 0.25% Trypsin-EDTA incubation at 37 °C for another 30 min. Then, CsCl was added into cell crude lysate at a final concentration of 0.472 g/mL and incubated at 37 °C for 30 min. The mixture was centrifuged at 3,500 rpm for 30 min, the clarified virus/CsCl solution was transferred into another tube and adjusted to a final density of about 1.40 g/mL. The final clear mixture was loaded into tubes and centrifuged at 41,000 rpm for 36 hrs at 20 °C using an TH641 rotor in a Sorvall™ WX (Thermo Scientific). After two rounds of CsCl banding, an aliquot (500 µL) of gradient fractions was collected using a Gradient Station (BioComp Instruments, Fredericton, N.B., Canada), determined for values of refractive index using an Abbe Refractometer, and quantified by qPCR for vector genomes. The peak fractions were dialyzed against PBS buffer.

For the HEK293 cell system, the rAAV2/HBoV1 vector was generated by co-transfection of pAAV2ITR-GFP-Luc, pCMVNS*Cap-P5Rep, and pHelper that expresses adenoviral E2, E4Orf6 protein and VA RNA [3] into twenty 150-cm^2^ plates of HEK293 cells (80% confluency) at a ratio 2:3:1, as previously described [42]. At 72 hrs post-transfection, cell pellets were collected and treated, and recombinant vector was purified as described above for infected Sf9 cells.

### 2.5. Western Blot and Southern Blot Analyses

Western blotting was performed as previously described [59]. For Southern blotting, low molecular weight (Hirt) DNA was extracted from BEV infected Sf9 cells, and the analysis was performed as previously described [60], using an AAV2 *cap* gene probe.

### 2.6. Quantitative Real Time PCR (qPCR) Analysis of rAAV2/HBoV1

The titers of rAAV2 and rAAV2/HBoV1 in DNase I digestion-resistant particles (DRP) were determined by a qPCR method that has been used previously [42]. Briefly, 50 μL aliquots of the samples were incubated with 20 units of Benzonase (MilliporeSigma) for 2 hrs at 37 °C, followed by 20 μL of proteinase K (15 mg/mL) at 56 °C for 10 min. Viral DNA was extracted using a QIAamp blood mini kit (Qiagen, Hilden, Germany), and then eluted in 50 μL of deionized water. A plasmid containing a GFP ORF was used to establish a standard curve for absolute quantification. The amplicon primers and dual-labeled probe were designed using Primer Express (Applied Biosystems, Foster City, CA, USA) and synthesized at IDT Inc. (Coralville, Iowa, USA). The sequences of the primers and probe specific to the GFP coding sequence are as follows: forward primer, 5′-CTG CTG CCC GAC AAC CA-3′; reverse primer, 5′-TGT GAT CGC GCT TCT CGT T-3′; and dual-labeled probe, 5′ 6FAM-TAC CTG AGC ACC CAG TCC GCC CT-3′ Iowa Black FQ. Probe qPCR MasterMix (Applied Biological Materials Inc., Vancouver, Canada) was used for qPCR following a standard protocol on a 7500 Fast Real-Time PCR System (Applied Biosystems, Foster City, CA, USA), and 2 μL of extracted DNA was used in a reaction volume of 20 μL.

### 2.7. Transmission Electron Microscopy

For each recombinant virus, aliquots of 50 µL in the peak fractions were performed for electron microscopy analysis in the Electron Microscopy Research Laboratory (EMRL) of the University of Kansas Medical Center. Briefly, two to five µL of each sample were spotted onto formvar-coated, carbon-stabilized, 200-mesh copper grids for 1 min and then washed with deionized water. Staining was achieved by adding five drops of 2% uranyl acetate. Excess staining was removed immediately by adsorption to filter paper, and the samples were then air dried. The grids were examined on a Transmission Electron Microscope (JEM-1400; JEOL, Peabody, MA, USA) at a magnification setting of 30,000 × and an accelerating voltage of 100 KV.

### 2.8. rAAV2/HBoV1 Transduction of HAE-ALI Cultures

We followed a previously described protocol to infect HAE-ALI cultures with rAAV2/HBoV1 [42]. Briefly, proteasome inhibitor doxorubicin and N-acetyl-l-leucyl-l-leucyl-l-norleucine (LLnL) at the final concentrations of 2.5 µM and 20 nM, respectively, were added into the culture medium of the basolateral chamber. Then, a total of 10^9^ DRP of rAAV2/HBoV1 in 50 µL of medium were applied directly onto the apical surface of the airway epithelia at an MOI of ~2000 DRP/cell. At 12 hrs post-infection, the medium and virus were removed from the apical surface, and the basal medium was replaced with fresh medium without addition of proteasome inhibitors.

### 2.9. Measurement of Luciferase Reporter Expression

Luciferase enzyme activity was examined using a Luciferase Assay System kit (Promega, Madison, WI, USA) following the manufacturer’s instructions. Briefly, HAE cells were collected after EDTA and trypsin treatments of the HAE-ALI cultures, and equal numbers of the cells from compared HAE-ALI cultures were transferred into wells of a 96-well plate. The wells were then added with 20 µL of 1× Lysis reagent, followed by mixing with 100 µL of Luciferase Assay Reagent and the light produced on a Synergy H1 microplate reader (Synergy H1, BioTek, Winooski, VT, USA) was measured.

### 2.10. Antibodies Used

A monoclonal antibody (clone 303.9) that reacts with AAV2 Rep78 and Rep52 and a monoclonal antibody (clone B1) that reacts with AAV2 Cap were purchased from American Research Products, Inc. (Waltham, MA, USA). Rat anti-HBoV1 Cap that reacts with VP1, VP2, and VP3 and rat anti-HBoV1 NP1 that recognizes the NP1 protein were made in house and have been described previously [26,31].

## 3. Results

### 3.1. Design of the Baculovirus Expression vector System

We used two BEVs, Bac-AAV2ITR-GFP and Bac-AAV2Rep-Cap (Figure 1E), to produce rAAV2 in Sf9 cells, which serves as a comparative control for the test of the production of the rAAV2/HBoV1 vector. In Bac-AAV2Rep-Cap, the AAV2 *rep* and *cap* genes were modified to allow expression of the Rep78 and Rep52 proteins and the VP1, VP2, and VP3 proteins from two species of mRNAs transcribed from Ph and P10 baculovirus promoters, respectively [56]. To generate the rAAV2/HBoV1 vector, we similarly made two BEVs, Bac-AAV2ITR-GFP-Luc, which harbors a 5.4-kb oversized rAAV2 genome, and Bac-AAV2Rep-HBoV1Cap (Figure 1A), which expresses AAV2 Rep78 and Rep52 under the Ph promoter and HBoV1 VP1, VP2 and VP3 under the P10 promoter. In addition, we made Bac-HBoV1NP1 that expresses the HBoV1 NP1 protein to look for a role of the NP1 in vector production.

We also compared the production and biologic properties of the rAAV2/HBoV1 vectors from insect cell and mammalian cell systems in parallel. To this end, we made the cis and trans plasmid constructs for rAAV2/HBoV1 vector production in HEK293 cells in the format similar to those used in the BEVs. The pAAV2ITR-GFP-Luc (5.4-kb) harbors the identical 5.4-kb rAAV2 genome that was in the transfer BEV Bac-AAV2ITR-GFP-Luc. The pCMVNS*Cap-P5Rep is the HBoV1 *cap* and AAV2 *rep* two-in-one expression plasmid, which expresses HBoV1 NS (NP1, NS3 and NS4) and Cap (VP1, VP2, and VP3) under the cytomegalovirus immediate early promoter (CMV) [27,28] and AAV2 Rep78 and Rep 52 under the AAV2 P5 and P19 promoters, respectively (Figure 1B).

### 3.2. Analyses of Protein Expression and Replication of the rAAV2 Genome in Sf9 Cells

**Figure 2:**
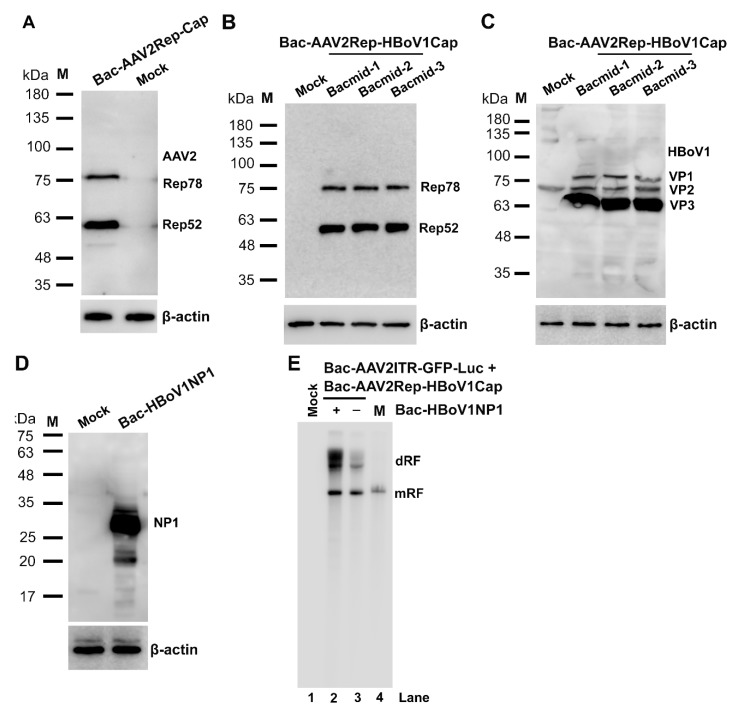
Expression of AAV2 Rep and Cap proteins and HBoV1 Cap and NP1 proteins in BEV-infected Sf9 cells. (**A**–**D**) Western blotting. Sf9 insect cells were infected with Bac-AAV2Rep-Cap (**A**), Bac-AAV2Rep-HBoV1Cap (**B**,**C**), or Bac-HBoV1NP1 (**D**). The infected cells were collected at 72 hrs post-infection and subjected to Western blot analysis. (**A**) AAV2 Rep proteins were detected with an anti-Rep monoclonal antibody. (**B**,**C**) Bac-AAV2Rep-HBoV1Cap generated from transfection of three bacmid (Bacmid-1-3) were infected with Sf9 cells independently. (**B**) AAV2 Rep proteins were detected with an anti-Rep monoclonal antibody, and (**C**) HBoV1 Cap protein expression was detected with an anti-HBoV1 Cap protein antiserum. (**D**) HBoV1 NP1 was detected with a rat anti-HBoV1 NP1 antiserum. β-actin served as a loading control. Mock, uninfected cells. (**E**) Southern blotting. Sf9 cells were infected with Bac-AAV2ITR-GFP-Luc and Bac-AAV2Rep-HBoV1Cap with (+) or without (-) co-infection of Bac-HBoV1NP1. Cells were collected at 72 hrs post-infection and subjected to extraction of lower molecular weight (Hirt) DNAs, which were analyzed by Southern blotting. Mock, uninfected Sf9 cells as a control; M, a marker of a rAAV2ITR-GFP-Luc proviral replicative form (RF) genome of 5.4 kb. dRF and mRF, double and monomer RF.

To characterize the expression of AAV Rep and Cap, Sf9 cells grown in suspension culture were infected with Bac-AAV2Rep-Cap, Bac-AAV2Rep-HBoV1Cap, and Bac-HBoV1NP1, respectively. The infected cells were collected at 72 hrs post-infection, and the expression of viral proteins was analyzed by Western blotting. We first examined the expression of AAV2 Rep from the Sf9 cells infected with Bac-AAV2Rep-Cap (Figure 2A) and Bac-AAV2Rep-HBoV1Cap (Figure 2B), respectively. The results of Western blotting showed that the expressions of the AAV2 Rep by the P10 promoter from these two BEVs demonstrated a similar pattern, which both expressed AAV2 Rep78 and Rep52 at a ratio close to ~1:2. Of note, when we constructed the Bacmid pBac-AAV2Rep-HBoV1Cap, the codon optimization of AAV2 rep (Figure 1C) was adopted from one mRNA transcript, which was used in Bac-AAV2Rep-Cap [56]. The HBoV1 Cap expression from the Sf9 cells infected with Bac-AAV2Rep-HBoV1Cap was also analyzed with Western blotting, which confirmed that the optimization of initiation codons (Figure 1D) led to the expression levels of HBoV1 VP1, VP2, and VP3 at a ratio close to ~1:1:10 (Figure 2C), similar to that was observed from the transfection of the pCMVNS*Cap in HEK293 cells [28]. The cryptic polyadenylation signals (pA) resided inside the unique sequence in the VP1 ORF, which serve as the proximal pA preventing HBoV1 *cap* transcription in mammalian cells in the absence of NP1 expression [28], appeared to not be effective in the insect cells. These results confirmed that the expression strategy that Bac-AAV2Rep-Cap utilized to express the overlapping genes *rep* and *cap* from the baculoviral promoters P10 and Ph was applicable for the construction of Bac-AAV2Rep-HBoV1Cap. This AAV2/HBoV1 trans helper possessed the same capability to express AAV2 Rep proteins and also HBoV1 VP1, VP2 and VP3, and more importantly, they were expressed at the expected ratios. Thus, one BEV was able to express the parvoviral proteins from different genera efficiently without mutual disruption.

To determine the function of HBoV1 NP1 during the replication of the rAAV2 genome, we made a Bac-HBoV1NP1. It expressed HBoV1 NP1 at ~25 kDa (Figure 2D). Then, Sf9 cells were co-infected with Bac-AAV2ITR-GFP-Luc and Bac-AAV2Rep-HBoV1Cap, with or without Bac-HBoV1NP1. The infected cells were sampled at 72 hrs post-infection and analyzed for the presence of rAAV2 replicative-form (RF) DNA intermediates by Southern blotting (Figure 2E). Although ssDNA was not obviously detected in both groups, clearly much more double replicative form (dRF) DNA was observed in the presence of NP1 expression (Figure 2E, compare lanes 2 vs 3). Although NP1 is not required to modulate the HBoV1 cap expression in Sf9 cells as it does in mammalian cells, it positively enhances the replication of rAAV2 genomes. The mechanism of NP1’s involvement in rAAV2 replication in Sf9 cells remains unclear.

### 3.3. rAAV2/HBoV1 vector is Successfully Produced in Sf9 Cells and NP1 Plays a Role in Increasing vector Yield

**Figure 3:**
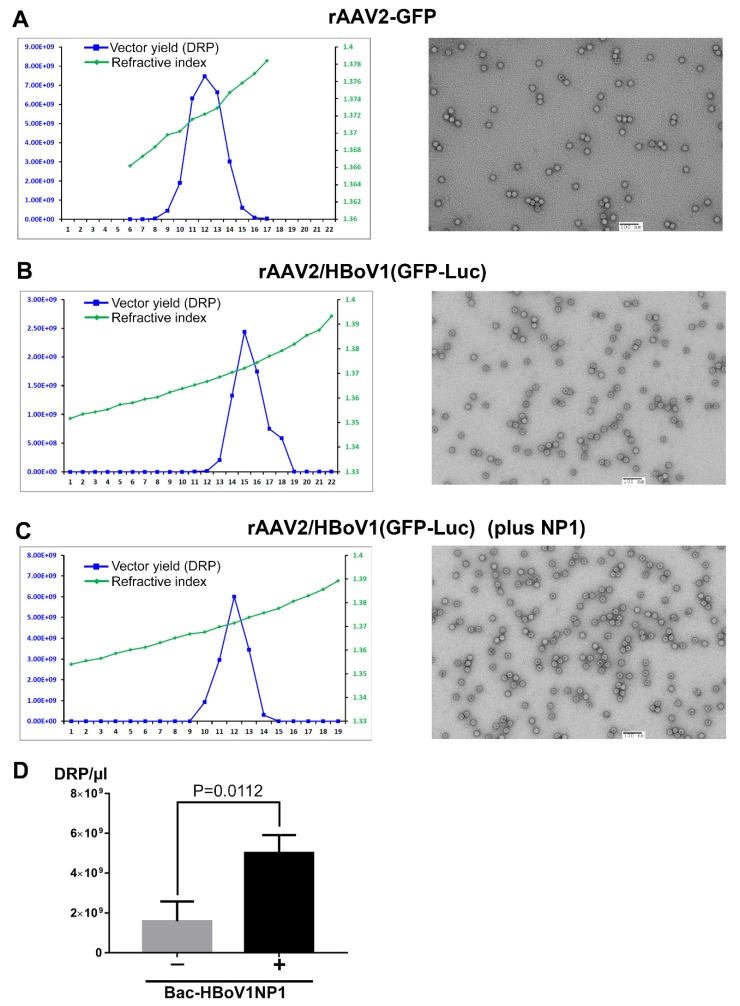
Purification of rAAV2 and rAAV2/HBoV1 vectors produced from BEV-infected Sf9 cells. (**A**–**C**) Vector production. Sf9 cells were co-infected with Bac-AAV2ITR-GFP and Bac-AAV2Rep-Cap (**A**), Bac-AAV2ITR-GFP-Luc and Bac-AAV2Rep-HBoV1Cap (**B**), or Bac-AAV2ITR-GFP-Luc, Bac-AAV2Rep-HBoV1Cap, and Bac-HBoV1NP1 (**C**). Cell lysates from infected cells were fractionated by CsCl equilibrium ultracentrifugation. Left panel: qPCR analysis was used to determine the DRP in each fraction of 0.5 mL (blue line); the density of each fraction was determined as refractive index and is shown by the line in green. Right panel: transmission electron micrographs of rAAV2 or rAAV2/HBoV1 vectors, which were negatively stained with a 1% uranyl acetate solution. Bar = 100 nm. (**D**) Comparison of rAAV2/HBoV1 production with or without NP1 expression in Sf9 cells. The experiments in panels B&C were repeated three times in parallel. Purified vectors at the peak fraction were quantified and compared. Averages and standard deviations are shown. Statistical analysis was performed to get the P value using student “t” test.

As a parallel control, we infected 200 mL of Sf9 cells with Bac-AAV2ITR-GFP and Bac-AAV2Rep-Cap for rAAV2 vector production. At 72 hrs post-infection, the infected cells were collected and lysed, and rAAV2 was purified by CsCl density gradient centrifugation. Fractions at a volume of 500 µL were collected and quantified for DRP by qPCR and demonstrated a peak at a refractive index of 1.372 (a density of ~1.40 g/mL) (Figure 3A, left). An electron micrograph of the rAAV2 produced is shown (Figure 3A, right) displaying particles of ~25 nm in diameter, a typical morphologic feature of AAV. In the peak fraction, the rAAV2 vector yield reached 7.52 × 10^9^ DRP/µL, indicating that the Sf9 system to produce rAAV vector was successful at a yield of ~1 × 10^4^ DRP/Sf9 cell from 200 mL Sf9 cells at a density of 2 × 10^6^ cells/mL, a total 4 × 10^8^ cells.

The production of the rAAV2/HBoV1 vector was performed with BEV infection to Sf9 cells under the same conditions for rAAV2. We compared two groups of BEVs with or without expression of HBoV1 NP1 in parallel by infecting 4 × 10^8^ cells of Sf9 cells: Group I, with Bac-AAV2ITR-GFP-Luc and Bac-AAV2Rep-HBoV1Cap; Group II, with Bac-rAAV2ITR-GFP-Luc, Bac-AAV2Rep-HBoV1Cap, and Bac-HBoV1NP1. At three days post-infection, the infected cells were collected and lysed, and vectors were purified by CsCl density gradient centrifugation. The refractive index and DRP of each fraction are shown at the left in Figure 3B,C, and the transmission electron microscopy demonstrated that the rAAV2/HBoV1 vector had a typical parvovirus icosahedral structure that was ~25 nm in diameter as shown at the right in Figure 3B,C. Without expression of HBoV1 NP1, an average vector yield was 1.6 × 10^9^ DRP/µL in the peak fraction of 500 µL; however, there was a significant increase to 5.0 × 10^9^ DRP/µL with the help of Bac-HBoV1NP1, confirming that expression of NP1 significantly increased vector yield by three times (Figure 3D). Notably, the expression of NP1 led to an increase in rAAV2 replicative-form (RF) DNA intermediates (Figure 2E), which could be responsible for the enhanced production.

### 3.4. Comparison of the Transduction Efficiencies Between rAAV2/HBoV1 vectors Produced in Sf9 Cells and in HEK293 Cells

**Figure 4:**
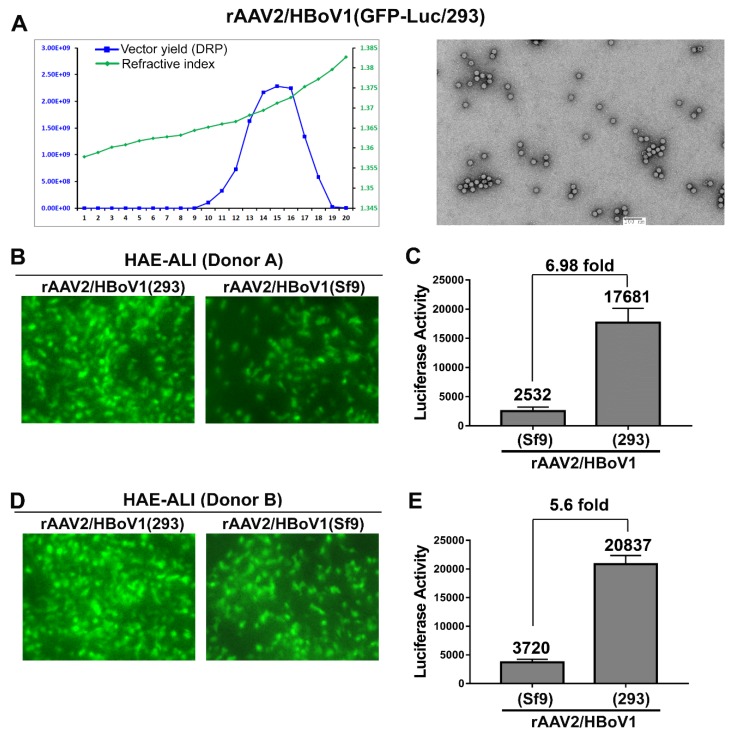
Comparison of the transduction efficiency between the rAAV2/HBoV1 vectors produced in Sf9 cells and HEK293 cells. (**A**) rAAV2/HBoV1 vector produced from HEK293 cells. HEK293 cells were transfected with pAAV2ITR-GFP-Luc, pCMVNS*Cap-P5Rep, and pHelper. Cell lysates from infected cells were fractionated by CsCl equilibrium ultracentrifugation. Left panel: qPCR analysis was used to determine the DRP in each fraction (blue line); the density of each fraction was determined as refractive index using an Abbe refractometer and is shown by the line in green. Right panel: a transmission electron micrograph of rAAV2/HBoV1(293) vectors. (**B**–**E**) rAAV2/HBoV1 transduction of HAE-ALI. HAE-ALI cultures prepared form Donor A (**B**,**C**) and Donor B (**D**,**E**) were transduced with rAAV2/HBoV1 either produced from Sf9 or HEK293 cells at an MOI of ~2000 DRP/cell from the apical surface. The rAAV2/HBoV1 vector was applied directly onto the apical surface of the airway epithelia. HAE cells were examined for GFP expression at 10 days post-transduction. Images were taken with an Eclipse Ti-S microscope (Nikon, Melville, NY, USA) at a magnification of × 20 (**B**&**D**). Luciferase activity was assayed at 10 days post-transduction (**C**&**E**). Averages and standard deviations generated from at least three independent experiments are shown. Statistical analysis was performed to get the P value using student “t” test.

It is encouraging that the yield of rAAV2/HBoV1 produced from the Sf9 cell system was comparable to that of rAAV2 in Sf9 cells in the presence of NP1 expression (5.0 × 10^9^ vs 7.5 × 10^9^ DRP/ul in the peak fraction of 500 µL). We next characterized its biological function in transducing HAE-ALI cultures. To this end and for fair comparison, we produced rAAV2/HBoV1(293) by transfection of pAAV2ITR-GFP-Luc, pCMVNS*Cap-P5Rep and pHelper into HEK293 cells of 20 × 145-mm plates and obtained a yield of 2.3 × 10^9^ DRP/µL at the peak fraction (Figure 4A). We apically infected the well-differentiated HAE-ALI cultures, which were generated from airway epithelial cells from two different donors, with equal amounts of vectors produced from Sf9 cells or HEK293 cells. Proteasome inhibitors LLnL and doxorubicin were only applied in the basal chamber during the infection period of 12 hrs [42]. At seven days post-infection, cells were examined for the GFP expression under a fluorescence microscope, and images were taken at the same setting (Figure 4B,D). We observed more green cells from the infection of rAAV2/HBoV1(293) with relatively stronger intensity of fluorescence than its counterpart infection transduced of the rAAV2/HBoV1(Sf9). Next, the cells were lysed for quantification of the luciferase activity (Figure 4C,E), which revealed that the rAAV2/HBoV1(293) vector has a transduction efficiency 5–7 times higher than the rAAV2/HBoV1(Sf9) vector.

## 4. Discussion

Cross-genera pseudopackaging between parvoviruses was first established by pseudotyping a rAAV genome into a capsid of human parvovirus B19 [61] for a chimeric AAV-B19 vector in HEK293 cells, which demonstrated high tropism to human erythroid cells. In 2013, we successfully packaged an rAAV2 genome into the capsid of HBoV1 in HEK293 cells, generating rAAV2/HBoV1 chimeric vector [42]. The rAAV2/HBoV1 vector has a high tropism for polarized human airway epithelia and is able to encapsidate an oversized rAAV2 genome of 5.8-kb, representing one of the best rAAV vectors for gene delivery to human airways and holding much promise for use in preclinical trials of CF gene therapy in ferrets and human trials of CF patients [62].

To meet the high demand of rAAV2/HBoV1 vector production at a large quantity, in this study, we took advantage of the rAAV2 vector production system in insect cells. We modified HBoV1 *cap* gene in Bac-AAV2Rep-HBoV1Cap that expressed VP1, VP2, and VP3 at a ratio of ~1:1:10 in Sf9 cells, and proved that the rAAV2/HBoV1 vector was produced in Sf9 cells. More importantly, with the co-infection of a BEV expressing HBoV1 NP1, the rAAV2/HBoV1 vector was produced at a yield of 5.0 × 10^9^ DRP/µL, an equivalent efficiency as that of the rAAV2 vector in Sf9 cells (7.5 × 10^9^ DRP/µL in the peak fraction) from a small suspension culture (200 mL of Sf9 cells at a density of 2 million/mL) (Table 1).

The yield of rAAV2/HBoV1 in Sf9 cells is ~10–100-fold higher than in HEK293 cells, considering a yield per cell [47,53,54,56]. The infection of the BEVs to Sf9 cell suspension is simpler than the plasmid transfection to HEK293 cells, and the process is easily scalable for large preparation, e.g., with a Bio-Reactor. It was previously reported that the biological characteristics of Sf9 cell-produced rAAV is equivalent to the HEK293 cell-produced rAAV [47,48,53,63]. However, in contrast, we observed that the transduction activity of the rAAV2/HBoV1(Sf9) vector produced from Sf9 cells is 5–7 times lower than that of the rAAV2/HBoV1(293) vector packaged in HEK293 cells (Figure 4C,E). We speculated that the rAAV2 genomes may not be as well packaged in HBoV1 capsids as that in AAV2 capsids, thus we examined these vector preps under transmission electron microscopy. We noticed that the rAAV2/HBoV1(293) vector barely had any empty particles (>95% full particles) (Figure 4A) as did the rAAV2 vector produced from Sf9 cells (Figure 3A, right panel), whereas rAAV2/HBoV1(Sf9) vectors had a high level of empty particles (only 50–60% full particles) (Figure 3B,C, right panels). Infection of BEV-Rep2Cap2, which was made following the Kotin strategy of Bac-AAV2Rep-Cap [56], expressed AAP in Sf9 cells, and knockout of the AAP decreased rAAV2 yield by 10 times [55]. This suggested that the AAP plays an important role in rAAV vector production in Sf9, which is likely through facilitation of the assembly of AAV capsids [22,23,24]. We currently do not know whether HBoV1 *cap* also expresses an AAP-like protein that may facilitate the assembly of the HBoV1 capsid, which warrants further investigation. Recently, glycosylation of rAAV has been reported and likely affects the potency of vector [64]. The possible variations in glycosylation between the vectors produced in HEK293 and Sf9 cells may also impact the transduction.

While the replication of the rAAV2 genome in Sf9 is not the rate limiting step for both rAAV2 and rAAV2/HBoV1 productions, it appears the trans functions for the pseudopackage of the rAAV2 genome in HBoV1 are less efficient than that for packaging it in the capsid of AAV2 or another AAV serotype. We have demonstrated an HBoV1 NS-free production system for rAAV2/HBoV1 in HEK293 cells [45]. In such a case, it appears that the expressions of AAV Rep proteins together with the helper components of adenovirus are sufficient for the cross-genera pseudopackage. It is clear that the adenovirus helper functions are not essential to the production of rAAV2 in Sf9 cells; however, it remains unknown whether they play a role in assisting the package of the rAAV2 genome into the HBoV1 capsid in HEK293 cells. Of note, the helper components from adenovirus are absent in the BEV system. Thus, the AAV2 Rep proteins, especially the AAV2 Rep52, might not be acting as efficiently in Sf9 cells as it does for pseudopackage of the rAAV2 genome in the HBoV1 capsid in HEK293 cells. For future improvement, we will tackle whether incorporation of one or more adenovirus helper components will solve this problem. Another consideration is the potent involvement of HBoV1 small NS proteins, despite the fact that the NS-free rAAV2/HBoV1 vector production system in HEK293 cells is against such action [45]. However, it is possible that they might confer necessary function in the absence of adenovirus function. Among them, the NS3 might be the first choice for the test, as it fully contains the helicase domain of the NS1, which is similar to the AAV2 Rep52 in structure [27] and executes helicase activity during viral genome packaging as the AAV2 Rep52 does [65].

In conclusion, we have established a rAAV2/HBoV1 vector production system in suspension culture of Sf9 cells for pseudopackage of the rAAV2 genome into the HBoV1 capsid. The yield of the rAAV2/HBoV1 vector is similar to that of rAAV2 produced in suspension Sf9 culture in a small volume, which is scalable in a large culture of Sf9 cells [49,50,51,53,66,67]. However, the current rAAV2/HBoV1-BEV system tends to produce more empty particles than the counterpart rAAV2 vector system. In the future, we will optimize the Sf9 cell production and purification system to reduce empty particles and to produce the rAAV2/HBoV1 vector in a large quantity as the suspension Sf9 cell culture can be easily scaled, which will enable the use of the vector for gene therapy of CF lung disease in large animal models.

## Figures and Tables

**Table 1:** Comparison of vector production in Sf9 vs HEK293 and with or without NP1 expression.

Vector	Helper *	Vector Yield in the Peak Fraction (500 µL)
rAAV2/HBoV1(Sf9)	None	1.6 × 10^9^ DRP/µL
rAAV2/HBoV1(Sf9)	Bac-HBoV1NP1	5.0 × 10^9^ DRP/ul
rAAV2(Sf9)	None	7.5 × 10^9^ DRP/µL
rAAV2/HBoV1(293)	pHelper	2.3 × 10^9^ DRP/µL

Note: 200 mL of Sf9 cells at a density of 2 × 10^6^ cells/mL (a total of 4 × 10^8^) and 20 145-mm plates of HEK293 cells (a total of 5 × 10^8^) were infected /transfected for rAAV vector production. Vectors in the peak fraction that has the density of 1.40 g/mL in CsCl were quantified after dialyzed. * Helper: other than *rep*/*cap* trans complementary.
